# Pennsylvania Military Surgeons

**Published:** 1861-07

**Authors:** 


PENNSYLVANIA MILITARY SURGEONS.—Professor Henry H. Smith, of this city, has been appointed by Governor Curtin Surgeon-General of Pennsylvania.

A few weeks ago, a Medical Board, consisting of Dr. Smith and Dr. Agnew, of Philadelphia, Dr. Dock, of Harrisburg, and Dr. King, of Pittsburg, met at the capital of the State for the purpose of examining applicants for admission into the Medical Staff of the Volunteer Service. It is said that 60 candidates were present, and that 230 permits had been issued.

A large number of young physicians of Pennsylvania have entered the Volunteer Service of the Army as surgeons and assistant surgeons. It is also understood that quite a number are applicants for admission into the Regular Army and Navy.

